# A dominant role of cell death in limiting Chandipura virus propagation at cell-saturating high multiplicity of infection

**DOI:** 10.1128/mbio.01013-26

**Published:** 2026-06-15

**Authors:** Bhawna, Swapnava Basu, Syed Yusuf Mian, Sanchi Arora, Yashika Ratra, Kasturi Ganguly, Sachendra S. Bais, Manidipa Banerjee, Abhyudai Singh, Soumen Basak

**Affiliations:** 1 Systems Immunology Laboratory, National Institute of Immunology, New Delhi, India; 2 Kusuma School of Biological Sciences, IIT Delhi 28817, New Delhi, India; 3 Department of Electrical and Computer Engineering, University of Delaware 5972, Newark, Delaware, USA; 4 Department of Biomedical Engineering, University of Delaware 5972, Newark, Delaware, USA; Indian Institute of Science, Bangalore, India

**Keywords:** Chandipura virus, multiplicity of infection, burst size, virus propagation, cell death, type-1 interferons, interferon-β, antiviral defense

## Abstract

Viruses transit from a low to high multiplicity of infection (MOI) regime in infected tissues. Type-1 interferons (IFNs) enforce a cellular state refractory to virus multiplication, while the death of infected cells eliminates viral replicative niche. Here, we investigated how these two innate antiviral mechanisms cooperate at various MOIs upon cell infection by Chandipura virus (CHPV), a cytopathic RNA virus implicated in several outbreaks of acute encephalitis in India. We found that as expected, a gradual increase in the input MOI from 0.02 to 2 led to a proportionate surge in the viral yield. Surprisingly, a further rise to MOI 20 caused a reduction in the progeny titer. Our mathematical modeling together with *ex vivo* infection studies involving knockout cells suggested that cell death—more so than virus-induced type-1 IFNs—restricted CHPV propagation at cell-saturating high MOIs, leading to a net fall in the yield at MOI 20. We argue that the distinct involvement of innate immune pathways at varied MOIs imparts robust cellular defense against cytopathic viruses.

**IMPORTANCE:**

After host invasion, a limited number of viruses initially provide for a low multiplicity of infection (MOI) of tissue cells. Ongoing viral multiplication allows for high multiplicity infection of host cells at a later stage. Type-1 IFNs and cell death constitute two principal arms of the antiviral cellular defense. We probed infection of cultured cells by Chandipura virus (CHPV), a virus implicated in encephalitis outbreaks, at various MOI. We find that CHPV propagation is restricted mainly through type-1 IFNs at low MOI and cell death at high MOI. Cell death not only destroys the viral replicative niche but also orchestrates pathological inflammation. In fact, moderating inflammation represents an important objective in the symptomatic management of viral diseases. While the proposed model needs to be tested for other viruses, our findings may bear significance for therapeutic intervention strategies and timing in viral diseases.

## INTRODUCTION

The intimately connected global civilization of the present era is also extremely vulnerable to epidemic threats. In particular, Chandipura virus (CHPV) has been linked to several outbreaks of acute encephalitis in India, including the most recent one in 2024 (1–4). Previous epidemiological studies captured a startling case fatality rate of 55% to 75% in CHPV outbreaks, with accompanying dysfunctions in the central nervous system, among children below 15 years of age (5). An improved understanding of molecular and cellular events driving pathogenesis during the course of CHPV infection may have a profound bearing on human health, particularly in the Indian subcontinent.

CHPV belongs to the Rhabdoviridae family and Vesiculovirus genera and possesses an enveloped negative-sense RNA genome (6, 7). A recent study implicated host-derived α-2-macroglobulin in viral entry into target cells although the exact identity of the cellular receptor for CHPV remains uncertain (8). Nevertheless, a series of prior investigations aptly defined the role of CHPV-encoded phosphoprotein and nucleocapsid protein in regulating the viral transcription-replication cycle in infected cells (9–12). Similarly, cellular factors that collaborate with viral matrix protein to promote CHPV budding from infected cells have been identified (13). Accordingly, the emphasis of CHPV research gradually shifted toward understanding host responses.

Type-1 IFNs and cell death constitute two principal arms of the innate antiviral cellular defense. Viral infection stimulates the nuclear translocation of RelA NF-κB heterodimers from their cytoplasmic IκBα-inhibited latent complexes and also activates Interferon regulatory factor 3 (IRF3). IRF3, in collaboration with RelA NF-κB, transcriptionally upregulates type-1 IFNs, including IFNβ. In autocrine and paracrine loops, type-1 IFNs secreted from infected cells then signal through the cognate IFN receptor (IFNR)—this triggers hundreds of interferon-stimulated genes (ISGs) (14). ISGs enforce an antiviral cellular state refractory for viral multiplication. Accordingly, genetic disruption of type-1 IFN signaling in *Ifnar1^−/−^* cells, which lack functional IFNR, was shown to enhance the multiplication of a number of RNA viruses, including CHPV (15–17).

Infection-inflicted cell death also limits viral multiplication, albeit by eliminating the viral replicative niche (18, 19). CHPV represents a cytopathic RNA virus. CHPV infection is associated with apoptosis driven by FAS-associated death domain (FADD)-mediated caspase 8 activation and also necroptosis involving receptor-interacting serine-threonine protein kinase 3 (RIPK3)-directed phosphorylation of mixed lineage kinase domain-like (MLKL) (16, 20). Not surprisingly, viruses tend to counteract the death of infected cells to ensure their continued growth. In fact, virus-activated RelA NF-κB also drives the expression of pro-survival genes, which prolong the life of infected cells. Correspondingly, a genetic deficiency of RelA NF-κB in mouse embryonic fibroblasts (MEFs) was shown to escalate the activation of apoptotic caspases and MLKL upon CHPV infection, leading to exacerbated cell death and a decrease in the viral yield (16).

Despite an in-depth understanding of the individual type-1 IFN and cell death pathways, how these two arms mutually cooperate remains less clear. In particular, previous studies documented that the effective multiplicity of infection (MOI) rises substantially within the tissue during the course of infection by animal and also plant viruses (21–23). Consistently, low MOI cell infection using influenza A virus revealed a gradual transition from low to high MOI settings (23). However, a plausible impact of altered input MOI on CHPV-induced type-1 IFN response and cell death, and also progeny yield, has not been examined.

Our combined experimental and mathematical studies identified a MOI-dependent collaboration between type-1 IFNs and cell death processes. We found that the type-1 IFN pathway was an important determinant of CHPV yield in the sub-saturating low MOI infection regime, whereas cell death represented a dominant antiviral mechanism during cell-saturating high MOI infections. In sum, our work suggests that distinct engagement of innate antiviral processes at varied MOIs instills robust cellular defense against cytopathic viruses.

## RESULTS

### A paradoxical relationship between viral input and yield at cell-saturating MOIs

**Fig 1:**
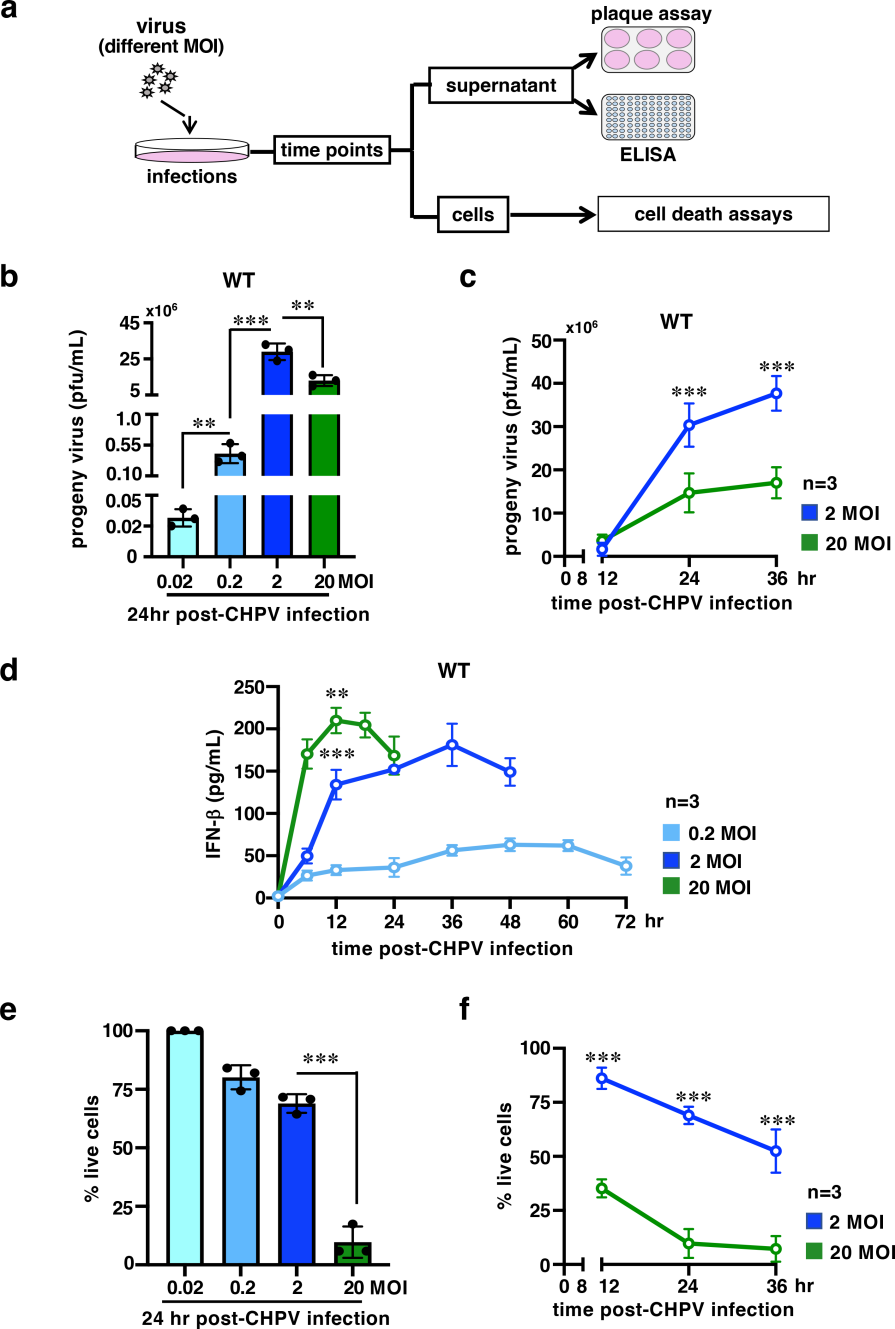
Comparing CHPV propagation in cultured cells infected at different MOIs. (**a**) Schema summarizing cell-infection experiments performed in this study. (**b**) Barplot revealing progeny virus titer in the culture supernatant of MEFs at 24 h post-infection with CHPV at the indicated input MOI. (**c**) Similarly, progeny virus yield was measured at 12 h and 36 h post-infection for cells infected at MOI 2 and 20. (**d**) ELISA showing accumulation of IFNβ in a time course in the culture supernatant of cells infected with CHPV at the indicated MOI. (**e**) CHPV-mediated cell death was measured at 24 h post-infection by crystal violet staining. The abundance of viable cells at various MOI was determined relative to corresponding uninfected MEFs and presented as bargraphs. (**f**) Infection-induced cell death was similarly measured at 12 h and 36 h post-infection. Data represent the means of three biological replicates ± SEM. The statistical significance was determined using two-way ANOVA test. **P ≤ 0.01; ***P ≤ 0.001.

To understand how the quantity of initial infecting particles impacts virus yield, we infected MEFs with CHPV at 0.02, 0.2, 2, and 20 MOI and determined the progeny titer in the culture supernatant by standard plaque assay (Fig. 1a). As expected, we recorded a proportionate rise in the titer, measured at 24 h post-infection, with a gradual increase in the input MOI from 0.02 to 2 (Fig. 1b). Unexpectedly, when the MOI was further raised from 2 to 20, we noticed a 2.2-fold reduction in the progeny yield. To ensure that our observation was not specific for a particular time point, we carried out a series of measurements at 12 h, 24 h, and 36 h post-infection, comparing CHPV yield at cell-saturating 2 and 20 MOIs. CHPV infection at MOI 20 produced, if anything, subtly more progeny virus at the early 12 h time point (Fig. 1c). We noticed a rise in the progeny yield in the cell culture supernatant with time for both MOIs. This increase was less remarkable for cell infection at MOI 20 than MOI 2, culminating to an overall deficit in the progeny titer at 24 h and 36 h time points for the MOI 20 regime. Next, we charted the innate antiviral cellular processes at various MOIs. We determined the abundance of IFNβ in the culture supernatant of infected cells by ELISA. Our analyses revealed that an increase in MOI both enhanced and accelerated peak IFNβ response. In particular, MOI 2 and MOI 20 infections produced an average peak IFNβ concentration of 181.07 pg/mL and 204.44 pg/mL in the culture supernatant at 36 h and 12 h post-infection, respectively (Fig. 1d). However, we noticed an early drop in the IFNβ production at 18 h post-infection in the MOI 20 regime. Using crystal violet staining, we then captured cell death as a function of input MOI. We found that increasing MOI caused a steady decline in the cell viability at 24 h post-infection, leading to 80% and 69% live cells at MOI 0.2 and MOI 2, respectively (Fig. 1e). Curiously, cell death was more strikingly escalated at MOI 20, leaving only a residual 9.7% live cells. Our time course analyses substantiated a significantly low live cell count at MOI 20 compared to MOI 2 as early as 12 h (Fig. 1f). We conclude that increasing input MOI at the sub-saturating regime augments CHPV yield while also intensifying type-1 IFN and cell death responses. At saturating MOIs, however, a further rise to high MOI values paradoxically plunges the progeny yield.

### Mathematical studies suggest distinct engagements of type-1 IFNs and cell death in limiting viral yield at varied MOI

Mathematical modeling provides valuable insights into the dynamic interplay between viral multiplication and host responses (24–26). In particular, previous ordinary differential equation (ODE)-based studies suggested that while IRFs tune subtype-specific type-1 IFN responses to influenza A virus, the extent of cell death could be a more accurate indicator of the severity of infections (27). Mathematical analyses also revealed that the duration, and not only the amplitude, of type-1 IFN feedback is crucial for eliminating infectious vesicular stomatitis virus (VSV) particles, particularly in the context of heightened viral replication rates (28). Similarly, combined single-cell studies and mathematical modeling experiments elucidated that an epigenetic-driven mechanism enforces transient heritability among the fraction of cells producing type-1 IFNs in a population (29). Our own mathematical studies identified that RelA NF-κB may promote CHPV yield by limiting infection-inflicted cell death (16). Although these investigations underscored the role of cell death and type-1 IFNs in viral growth, how input MOI impacts these dynamic relationships has not been mathematically dissected.

**Fig 2:**
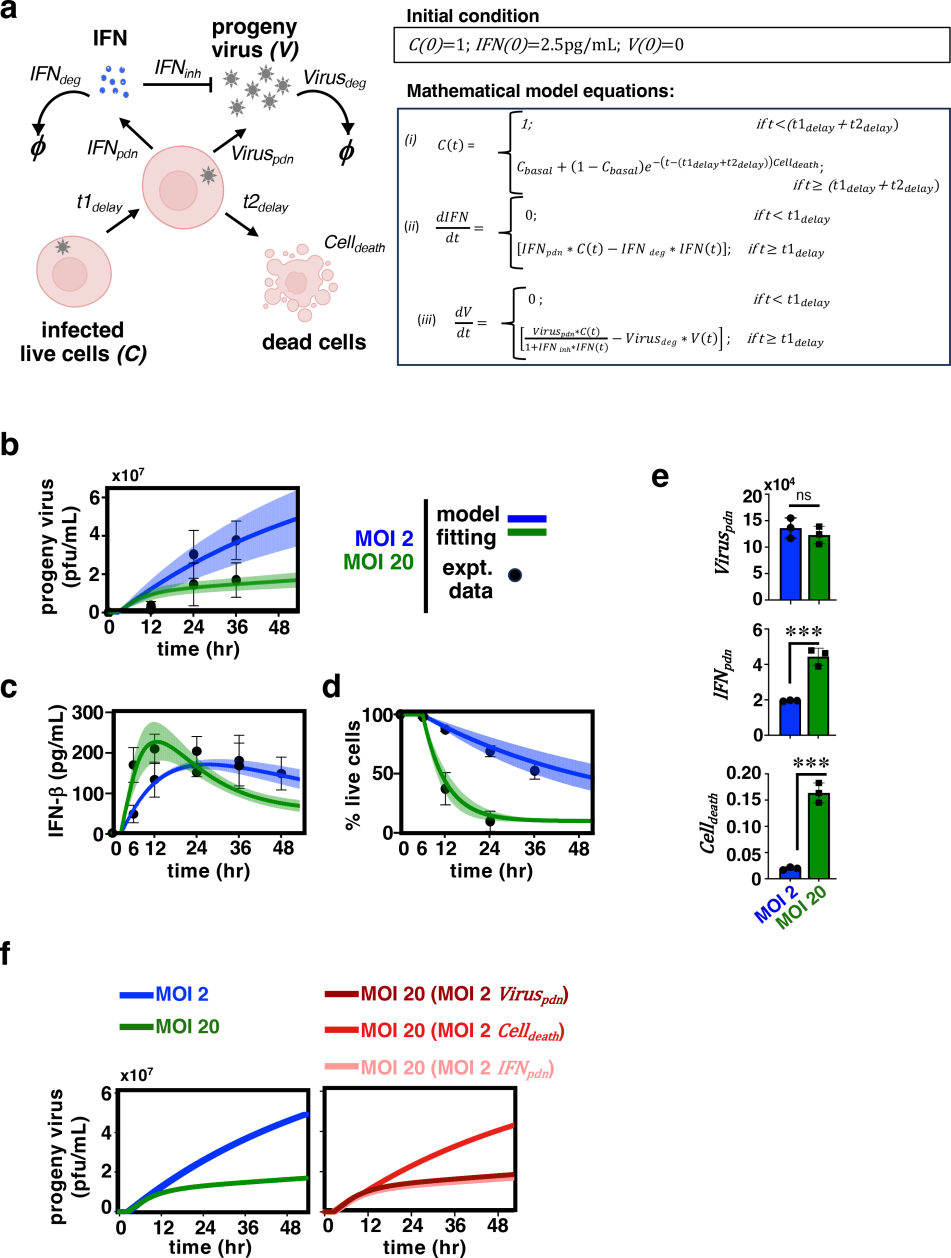
Mathematically probing CHPV multiplication and host responses at cell-saturating MOIs. (**a**) A cartoon depicting key processes involved in CHPV perpetuation at cell-saturating MOIs that were considered for mathematical analyses. Kinetic rate parameters associated with each of the reactions have been indicated. Right, a set of mathematical equations used for describing the relationship between number of infected live cells, IFN concentration, and progeny virus titer has been indicated. IFN(*t*) and *V*(*t*) have been used to represent IFN concentration in pg/mL and viral titer in pfu/mL at time *t*. The fraction of live, infected cells at time *t* has been described as *C*(*t*). Other parameters have been described in Table 1. (**b**–**d**) Fitting mathematical equations with experimental time course data related to progeny virus titer (**b**), cell-produced IFNβ levels (**c**), and infection-inflicted cell death (**d**) observed at MOI 2 and MOI 20. Experimental data points derived from biological replicates are indicated in black circles, and solid lines represent the fitted mean values for viral titer, IFN-β levels, or fraction of live cells. Shaded regions around the solid lines indicate the corresponding 95% confidence intervals. (**e**) Bar chart comparing the values of cell_death_, IFN_pdn_, and virus_pdn_ at MOI 2 and MOI 20 extracted from fitting exercise. Values of these rate parameters were independently determined using data from experimental replicates. Unpaired *t*-test was performed to determine the statistical significance. (**f**) Simulating virus yield as a function of time in the MOI 20 regime using the virus_pdn_*,* IFN_pdn_*,* or cell_death_ rate constant values linked to MOI 2. Data represent the means of three biological replicates ± SEM. ***P ≤ 0.001; ns, not significant, ≥0.05.

Because our experiments revealed a discrepant decrease in the progeny titer upon increasing the input MOI from 2 to 20, we set out to mathematically dissect CHPV growth. To this end, we developed a mathematical model to describe CHPV multiplication in cultured cells infected at saturating MOIs (Fig. 2a, equations i to iii. Productive viral infection of target cells at different MOIs follows a Poisson distribution, which posits 87% and 99.99% cells being effectively infected at MOI 2 and MOI 20, respectively. For the sake of simplicity, we approximated that all cells were infected at the onset of the simulation for both the MOIs. After a time delay of *t*1_delay_, infected cells produced IFNs involving a rate constant IFN_pdn_ and progeny viruses were generated involving a rate constant of virus_pdn_. We also considered a basal IFN level of 2.5 pg/mL in our model, as experimentally determined in WT cells (Fig. 1d; Table 1). After an additional delay of *t*2_delay_, cell death occurred involving a rate constant of cell_death_. IFN_inh_ was used as the rate constant for IFN-mediated inhibition of virus production by infected cells. The degradation rate of IFNs and the rate for inactivation of viruses were represented using IFN_deg_ and virus_deg_, respectively. Subsequently, we fitted our model to experimental time course data (Fig. 2b through d) for progeny virus titer (Fig. 2b), cell-produced IFNβ levels (Fig. 2c), and infection-inflicted cell death (Fig. 2d). From the fitting exercise, we then deduced *t*1_delay_ and *t*2_delay_ values to be 2.5 h and 3.5 h, respectively, regardless of the input MOI (see Materials and Methods). Similarly, we estimated the values for IFN_deg_, virus_deg_*,* and IFN_inh_ to be 8 × 10^−3^ h^−1^, 1.0 × 10^−4^ h^−1^, and 1.0 × 10^−4^ mL pg^−1^, respectively. The extracted values for virus_pdn_, IFN_pdn_*,* and cell_death_ at MOI 2 were 14.0 × 10^4^ pfu mL^−1^ h^−1^, 1.9 pg mL^−1^ h^−1^, and 0.02 h^−1^, respectively. To assess the reliability of the extracted parameters, we conducted an extensive literature survey of the closely related virus VSV (30–37). We observed that most of the parameter values assigned for WT MEF infection by CHPV in our model fell within an order of magnitude of those inferred from independently carried out VSV infection studies in MEFs (Table 1). In particular, the parameters virus_pdn_, IFN_pdn_, and cell_death_—each otherwise ascribed cell-type and MOI-specific distinct values in our study—were found to be well within the range of previous experimental determination. We further determined that while leading to a rather insignificant alteration in virus_pdn_, a change in the input MOI from 2 to 20 caused a 2.3-fold rise in IFN_pdn_ (Fig. 2e; Table 2). Interestingly, our mathematical formalism projected a marked 8-fold increase in cell_death_ from 0.02 h^−1^ to 0.16 h^−1^ when MOI was changed from 2 to 20 (Fig. 2e; Table 2). Taken together, a rise in the input MOI prompted heightened type-1 IFN response and aggravated cell death. We also noted that despite MOIs of 2 and 20 are expected to produce largely similar frequencies of infected cells initially, MOI 20 infection led to a markedly increased cell death. Accordingly, we postulate that in the cell saturating MOI regime, infection above a critical MOI threshold, and not the frequency of infected cells *per se*, triggers pervasive cell death.

**TABLE 1:** A chart describing the list of rate parameters and their corresponding values determined by fitting experimental data into the mathematical model^*a*^

Parameter	2 MOI			20 MOI			Defined values	Comments	Reference(s)
	WT	*Ifnar* ^−/−^	*Nfkbia* ^−/−^	WT	*Ifnar* ^−/−^	*Nfkbia* ^−/−^			
IFN(0) (pg mL^−1^ (basal)	2.5	2.5	5.25	2.5	2.5	5.25	~10	Data were generated in WT MEFs	(34)
*t*1_delay_ (h)	2.5	2.5	2.5	2.5	2.5	2.5	~2.5–5 h	Data were generated using VSV	(35)
*t*2_delay_ (h)	3.5	3.5	3.5	3.5	3.5	3.5	~1.5–4.5 h	VSV	(30)
Virus_pdn_ (pfu mL^−1^ h^−1^)	**14**.**0 × 10^4^ ([9.2–18.8] × 10^4^)**	**35.9 × 10^4^ ([26.4–45.4] × 10^4^)**	**0.17 × 10^4^ ([0.11–0.23] × 10^4^)**	**12.7 × 10^4^ ([8.5–16.9] × 10^4^)**	**43.7 × 10^4^ ([31.8–55.5] × 10^4^)**	**1.6 × 10^4^ ([0.2–3.0] × 10^4^)**	~10^4^–10^5^	VSV, MEFs	(33)
IFN_pdn_ (pg mL^−1^ h^−1^)	**1.98 (1.94–2.02)**	**0.79 (0.59–0.99)**	**0.28 (0.22–0.34)**	**4.51 (3.29–5.73)**	**2.74 (1.92–3.56)**	**0.70 (0.61–0.79)**	~1	VSV, MEFs	(36)
Cell_death_ (h^−1^)	**0.02 (0.014–0.026)**	**0.06 (0.045–0.075)**	**0.01 (0.003–0.017)**	**0.16 (0.12–0.20)**	**0.38 (0.26–0.50)**	**0.04 (0.032–0.048)**	0.02	VSV, MEFs; VSV, glioblastoma	(32, 33)
IFN_deg_ (h^−1^)	8 × 10^−3^	8 × 10^−3^	8 × 10^−3^	8 × 10^−3^	8 × 10^−3^	8 × 10^−3^	~ 8 × 10^−2^	VSV, MEFs	(36)
Virus_deg_ (h^−1^)	10^−4^	10^−4^	10^−4^	10^−4^	10^−4^	10^−4^	10^−3^	VSV, BHK21	(37)
IFN_inh_ (mL pg^−1^)	10^−4^	0	10^−4^	10^−4^	0	10^−4^	3.5 × 10^−3^	VSV, MEFs	(31)
*C*_basal_ (fraction of alive infected cells)	0.1	0.1	0.1	0.1	0.1	0.1	Based on our own experiments		

^*a*^ The 95% CI is shown in parentheses. Parameters shown in bold adopted distinct values depending on the genotype and multiplicity of infection (MOI).

**TABLE 2:** Comparing genotypes for MOI-dependent changes in the key rate parameters

Parameter	Fold change for changing MOI from 2 to 20		
	WT	*Ifnar1* ^−/−^	*Nfkbia* ^−/−^
Virus_pdn_ (pfu mL^−1^ h^−1^)	0.9, *P* ≥ 0.05, ns	1.2, *P* ≥ 0.05, ns	9.0, *P* ≤ 0.05
IFN_pdn_ (pg mL^−1^ h^−1^)	2.3, *P* ≤ 0.001	3.4, *P* ≤ 0.001	2.5, *P* ≤ 0.001
Cell_death_ (h^−1^)	8, *P* ≤ 0.001	6.3, *P* ≤ 0.001	4, *P* ≤ 0.001

To further understand how these kinetic rates may have influenced the viral yield at MOI 20, we charted virus generation over time in our mathematical model, albeit substituting MOI 20 rate values with those for MOI 2 (Fig. 2f). Our mathematical analyses revealed that neither MOI 2-associated virus_pdn_ nor MOI 2-associated IFN_pdn_ restored the virus titer in the MOI 20 regime. However, replacing the MOI 20 value with that of MOI 2 for cell_death_ alone almost entirely rescued the yield at MOI 20, leading to a progeny titer comparable to the MOI 2 regime (Fig. 2f). Our mathematical studies argue that escalating cell death, more so than elevated type-1 IFN production, restricts viral yield upon increasing input MOI in the cell-saturating MOI regime.

### Type-1 IFN deficiency fails to rescue the progeny yield at very high MOI

**Fig 3:**
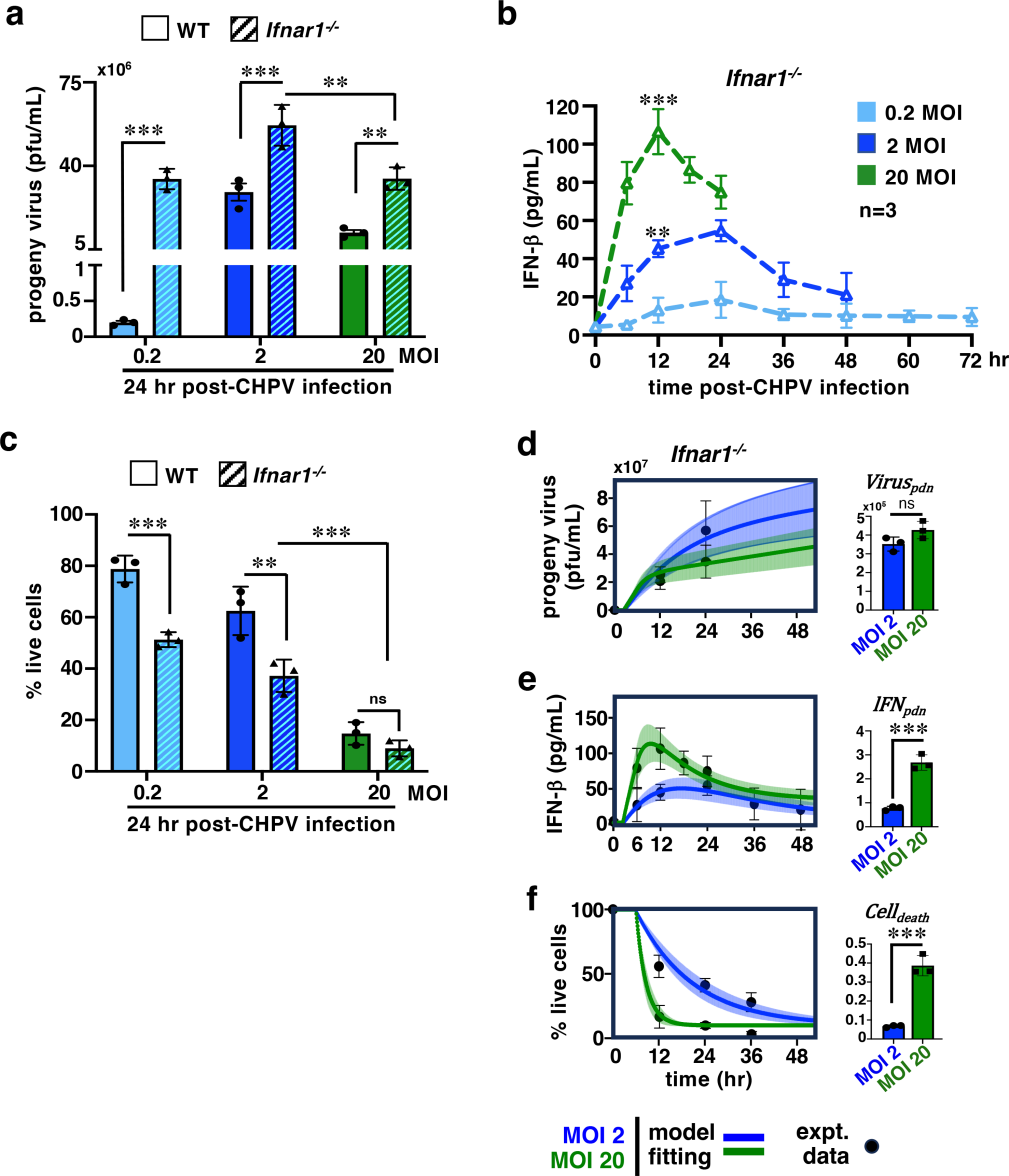
Charting CHPV growth in *Ifnar1^−/−^* MEFs infected at different MOI. (**a**) Barplot comparing virus yields in WT and *Ifnar1^−/−^* MEFs determined at 24 h post-infection at the indicated MOIs. (**b**) ELISA revealing the abundance of IFNβ in the culture supernatant of *Ifnar1^−/−^* cells infected with CHPV at the indicated MOIs. (***c***) Barplot showing cell death in WT and *Ifnar1^−/−^* MEFs at 24 h post-infection with CHPV at the indicated MOIs. (**d**–**f**) Line plots depicting fitting of mathematical equations with experimental time course data on CHPV propagation in *Ifnar1^−/−^* cells (left). Shaded regions represent 95% confidence intervals. Extracted values for various rate parameters are indicated in the accompanying bar chart (right). Data represent the means of three biological replicates ± SEM. The statistical significance was determined using two-way ANOVA test for panels a through c and using unpaired *t*-test for panel d **P ≤ 0.01; ***P ≤ 0.001; ns, not significant, ≥0.05.

To experimentally verify the predictions from our mathematical analyses, we examined CHPV infections in *Ifnar1^−/−^* cells, which lack functional type-1 IFN signaling. As such, type-1 IFN deficiency boosted viral yield, leading to a ~170-fold enhancement in the progeny titer at 24 h post-infection in *Ifnar1^−/−^* cells in the MOI 0.2 regime (Fig. 3a). Notably, this IFN phenotype was considerably less profound at saturating MOIs, resulting in merely 2-fold and 2.8-fold differences in the viral titer between WT and *Ifnar1^−/−^* cells at MOI 2 and MOI 20, respectively. We also noted that raising input MOI from 2 to 20 caused a reduction in the progeny yield in *Ifnar1^−/−^* cells similar to those observed in WT MEFs. Likewise, increasing MOI led to a rise in the IFNβ level in the culture supernatant of *Ifnar1^−/−^* cells, reaching a peak concentration of 106.5 pg/mL at 12 h post-infection in the MOI 20 regime (Fig. 3b). However, the peak value for IFNβ was somewhat lower in *Ifnar1^−/−^* MEFs than WT cells for a range of MOIs (compare Fig. 1d and 3b). *Ifnar1^−/−^* cells also displayed slightly diminished viability as opposed to WT MEFs at MOI 0.2 that otherwise showed MOI-dependent deterioration, sparing less than 9% lives cells at 24 h post-infection in the MOI 20 regime (Fig. 3c and d). We then fitted experimental time course data from *Ifnar1^−/−^* cells infected at saturating MOIs to our mathematical model and extracted various rate parameters for understanding IFN-mediated virus control (Fig. 3d through f; Table 1). Because these cells lacked type-1 IFN signaling, IFN_inh_, the rate constant representing IFN-mediated inhibition of virus production, was set to zero. Otherwise, the values for *t*1_delay_, *t*2_delay_, IFN_deg_, and virus_deg_ were deemed to be identical to those for WT. Our mathematical analyses disclosed that type-1 IFN deficiency led to a 2.6-fold to 3.5-fold increase in virus_pdn_ and another 2.3- to 3.0-fold increase in cell_death_, while resulting in a modest 1.6-fold to 2.5-fold reduction in IFN_pdn_ in these MOIs (Table 1). Akin to WT cells, changing MOI from 2 to 20 within the *Ifnar1^−/−^* settings produced a negligible change in virus_pdn_ (Fig. 3d), a moderate 3.4-fold increase in IFN_pdn_ (Fig. 3e), and a prominent 6.3-fold rise in cell_death_ (Fig. 3f) (also see Table 2). We infer that type-1 IFNs play only a minor role in MOI-dependent virus control, and in infection-induced cell death, at cell-saturating MOIs.

### Resilience to cell death improves the progeny CHPV yield at very high input MOI

Next, we asked if restraining virus-induced cell death bettered the progeny yield at high MOI in our experiments, as prescribed by our mathematical model. In addition to immune genes, RelA NF-κB dimers promote the expression of pro-survival factors, including cIAPs, cFLIP, Bcl2, and Bcl-XL (38). Our previous investigation demonstrated that the genetic absence of RelA aggravates cell death upon CHPV infection (16). In resting cells, RelA NF-κB factors are sequestered in the cytoplasm in a latent form by the inhibitory IκB proteins, the major isoform being *Nfkbia*-encoded IκBα (39). It was shown that disruption of IκBα-mediated inhibition promotes basally elevated NF-κB activity in *Nfkbia^−/−^* cells (40) and that constitutive NF-κB activity confers protection against death-inducing stimuli. Accordingly, we set out to examine *Nfkbia^−/−^* MEFs to assess the role of cell death in CHPV propagation (41).

**Fig 4:**
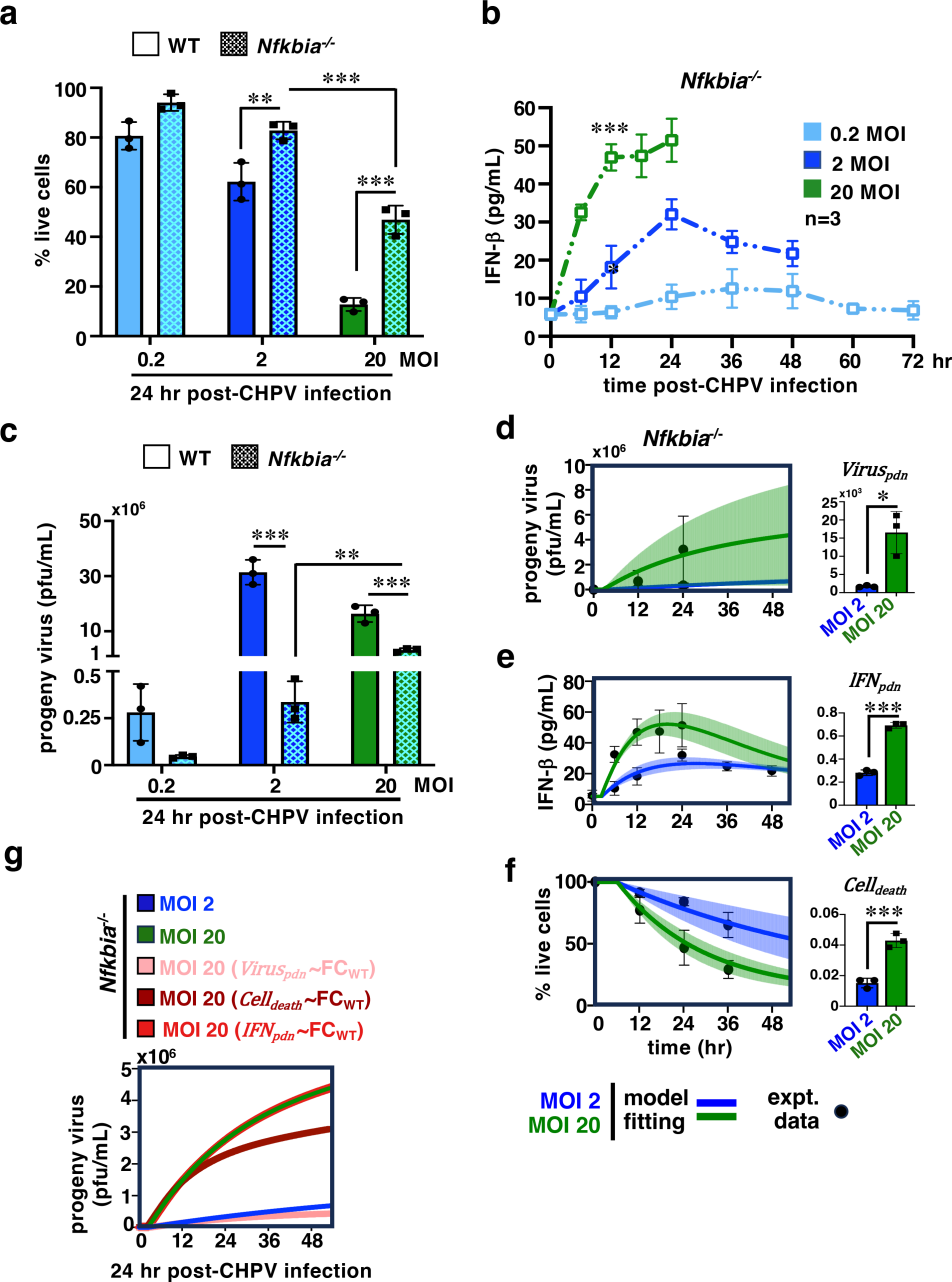
Studying CHPV propagation in *Nfkbia^−/−^* MEFs infected at different MOI. (**a, b, c**) Comparing WT and *Nfkbia^−/−^* MEFs infected at various MOIs for cell death (**a**), IFNβ production (**b**), and CHPV yield (**c**). (**d**–**f**) Fitting experimental time course data on CHPV propagation in *Nfkbia^−/−^* cells in our mathematical model (left). Shaded regions represent 95% confidence intervals. Extracted values for various rate parameters are also indicated (right). (**g**) Line plot charting the simulated progeny virus titer estimated in the *Nfkbia^−/−^* settings at MOI 20 using altered virus_pdn_, cell_death_, and IFN_pdn_ values that preserved the quantum of changes in these rates upon raising MOI from 2 to 20 to those determined in WT cells. Data represent the means of three biological replicates ± SEM. The statistical significance was determined using two-way ANOVA test for panels a through c and using unpaired *t*-test for panel d. *P ≤ 0.05; **P ≤ 0.01; ***P ≤ 0.001.

We found that IκBα deficiency, indeed, improved the cell viability, particularly more discernably at cell-saturating MOIs (Fig. 4a). Enhanced viability of *Nfkbia^−/−^* cells at MOI 20 led to a net 3.6-fold increase in the live cell count at 24 h post-infection compared to those in WT MEFs. Furthermore, *Nfkbia^−/−^* cells maintained ~2.2-fold excess IFNβ in the culture supernatant basally, while generating less IFNβ upon infection for a range of MOIs (Table 1, also compare Fig. 1d and 4b). Although increasing MOI elevated IFNβ levels, *Nfkbia^−/−^* cells did not display early termination of IFNβ production at MOI 20, as observed in other genotypes. These knockouts also produced substantially fewer progeny virus particles at varied MOIs (Fig. 4c). Importantly, a lack of IκBα arrested the decline in the virus yield upon raising the input MOI in the cell-saturating regime, instead leading to an overall 9.5-fold enhancement in the progeny titer at MOI 20 compared to MOI 2 (Fig. 4c). Fitting *Nfkbia^−/−^* cell data obtained at saturating MOIs to our mathematical model, we then deduced the relevant rate parameters (Fig. 4d through f; Table 1). Our mathematical approach disclosed that IκBα deficiency led to a substantial 82.3-fold and 7.9-fold drop in virus_pdn_ at MOI 2 and MOI 20, respectively, and an approximately 6.6-fold reduction in IFN_pdn_ (Table 1). Compared to WT, *Nfkbia^−/−^* cells also displayed a 2-fold decrease in cell_death_ at MOI 2, and a more prominent 4-fold fall at MOI 20 (Table 1). We then focused on probing how MOI changes from 2 to 20 altered these rates within the *Nfkbia^−/−^* settings. Despite an overall decline in the virus yield compared to WT MEFs, we captured a marked 9-fold elevation in virus_pdn_ in *Nfkbia^−/−^* cells when MOI was raised to 20 (Fig. 4d; Table 2). Notably, an equivalent increase in MOI led to a rather insignificant change in virus_pdn_ in WT cells. While MOI-dependent changes in IFN_pdn_ were analogous between WT and *Nfkbia^−/−^* MEFs, IκBα deficiency restricted the rise in cell_death_ upon increasing MOI to 4-fold compared to 8-fold in WT cells (Fig. 4e and f; Table 2). We then altered the MOI 20 values for virus_pdn_, cell_death_, and IFN_pdn_ in the *Nfkbia^−/−^* settings to achieve fold differences between MOI 2 and 20 mirroring those determined for WT cells. Our modeling studies revealed that restoring the quantum of changes in virus_pdn_ lessened the CHPV yield in *Nfkbia^−/−^* cells at MOI 20 to those determined at MOI 2 (Fig. 4g). Importantly, altering cell_death_ also diminished substantially the progeny titer, whereas IFN_pdn_ had no impact. Therefore, a rise in the virus production rate and improved cell viability both appear to play a role in augmenting the CHPV yield with increasing input MOI in *Nfkbia^−/−^* cells in the cell-saturating regime.

### Reduced cytopathicity supports increased progeny virus yield with rising MOI

**Fig 5:**
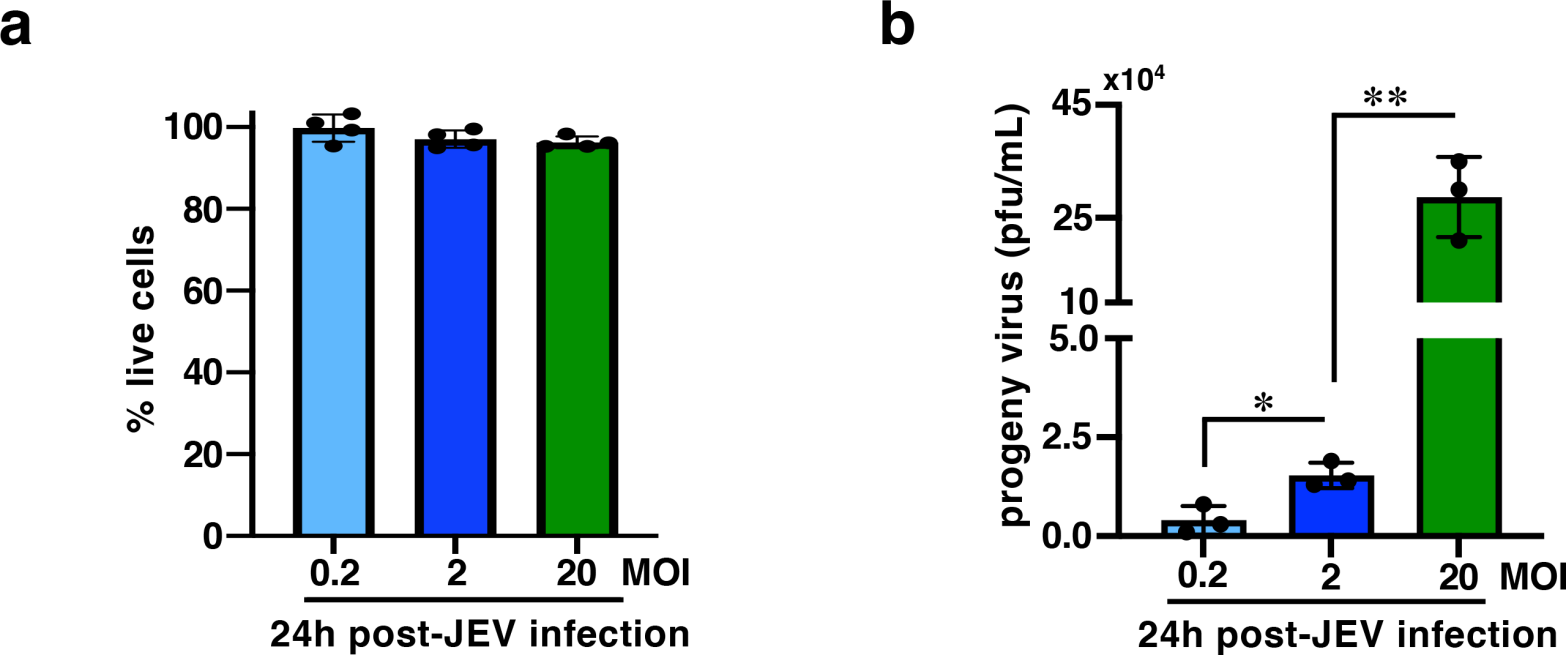
Investigating JEV infection of cultured cells at different MOIs. (**a**) Barplot showing cell death at 24 h post-infection for WT MEFs infected with JEV at the indicated MOIs. (**b**) Barplot comparing the progeny JEV virus yield in WT MEFs determined at 24 h post-infection for cell infections at the indicated MOIs.

Next, we asked whether infection by less cytopathic viruses would support a continued increase in viral yield with rising MOI in the cell-saturating MOI regime. Japanese encephalitis virus (JEV) is an enveloped, positive-sense RNA virus. Not unlike CHPV, JEV also compromises the blood-brain barrier, leading to acute encephalitis in infected subjects (42). As such, JEV infection induces rapid apoptotic death of neurons *in vitro*; JEV-mediated neuronal death is thought to underlie cerebral inflammation observed in patients (43, 44). Interestingly, we previously observed that JEV produces only a minor cytopathic effect upon infecting MEFs at MOI 2 (16). Accordingly, we set out to determine how the reduced cytopathicity associated with JEV infection of MEFs impacts MOI-dependent changes in the progeny yield. We infected MEFs with JEV at MOIs of 0.2, 2, and 20 and scored cell death at 24 h post-infection. Indeed, JEV induced negligible cell death at MOI 0.2 that was only minimally elevated even at MOI 20 (Fig. 5a). Importantly, an absence of aggravated cell death upon JEV infection of MEFs resulted in a gradual increase in the progeny yield upon rising MOI from 0.2 to 2 to 20 (Fig. 5b). Taken together, these findings indicate that the cytopathicity of a virus in a given cell type is a key determinant of progeny yield at high MOI. Nonetheless, we conjecture that even less cytopathic viruses may eventually display a decline in the progeny yield with further rise in MOI, albeit above a higher MOI threshold.

## DISCUSSION

**Fig 6:**
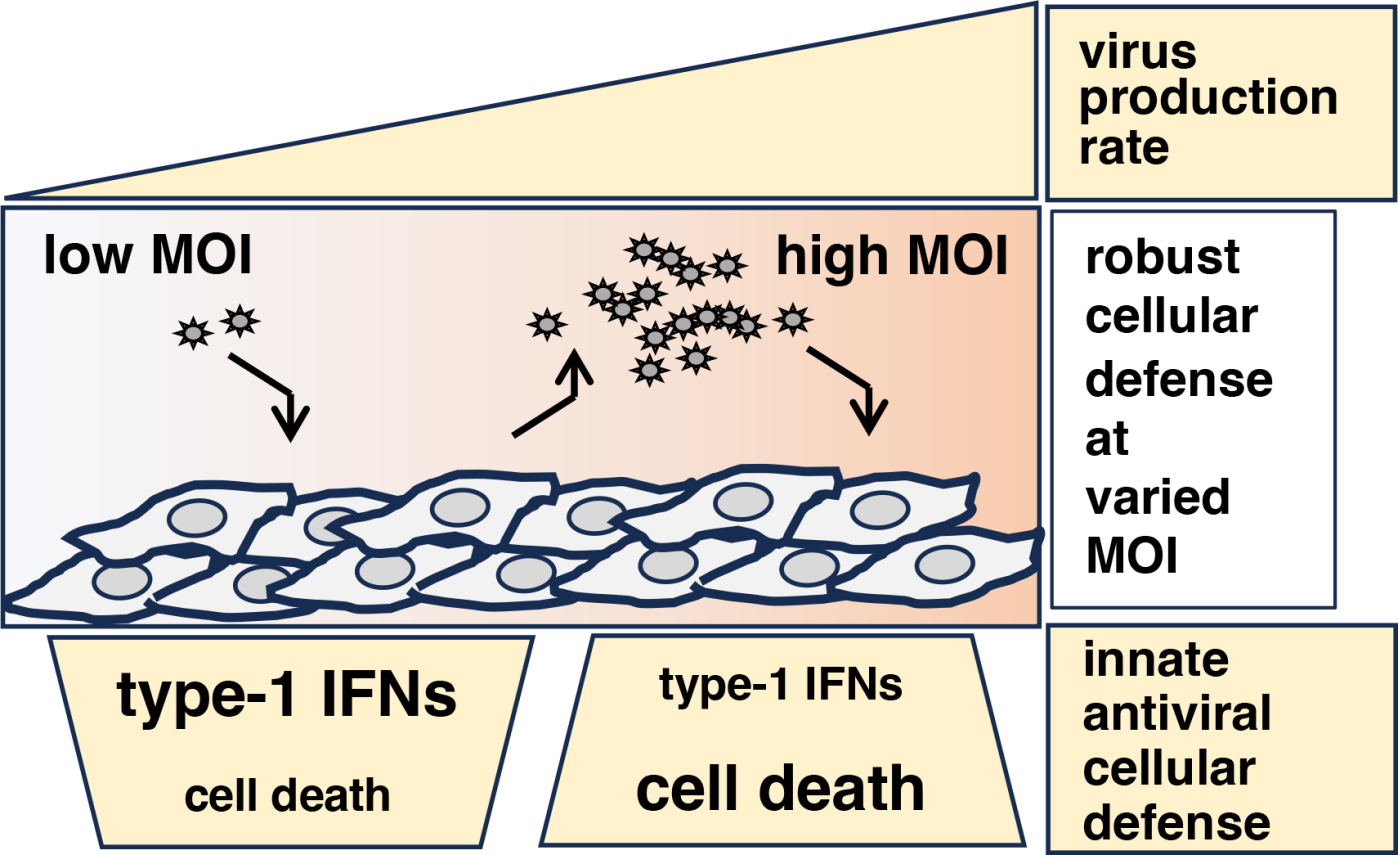
A model figure. A schema depicting the proposed collaboration between type-1 IFNs and cell death processes in limiting viral propagation at varied MOI.

At early time points during pathogenesis, a limiting number of invading viruses present in the target tissue provides for sub-saturating low MOI cell infections. In the sub-saturating regime, viruses spread from infected to neighboring uninfected cells for propagation. Gradual accumulation of viruses in the infected tissue percolates into a transition to the cell-saturating high MOI regime at a later time point (21–23). Combining experimental and mathematical studies, we determined the impact of input MOI on CHPV growth and antiviral cellular responses. As expected, increasing MOI boosted viral yield at sub-saturating low MOIs in our experiments, with type-1 IFNs playing a critical role in virus control (Fig. 6). In the cell-saturating high MOI regime, where cell-to-cell viral transmission is negligible, cell death played a dominant role in restricting CHPV multiplication, leading to a paradoxical drop in viral yield upon increasing MOI (Fig. 6). In a similar line, previous efforts to mathematically model influenza virus growth indicated that limiting cellular resources and virus-induced apoptosis curtail viral production at high MOI (45, 46). Concordantly, Rüdiger et al. found that adjusting the apoptosis rate of influenza virus-infected cells improves the model performance in the high MOI settings (23). These mathematical studies also predicted that multiple infections of cells, typically achieved at high MOIs, may undermine the antiviral state generated by type-1 IFNs (47). Our present study reinforced this conceptual framework involving experimental evidence obtained using mutant cells, enabling us to put forward a model where cell death curbs viral propagation mainly at high MOIs (Fig. 6). Therefore, our study charts a shift in antiviral cellular defense—from a predominant dependence on type-I IFN signaling at lower MOI to a critical reliance on cell death at high MOI. Our investigation captured multifaceted type-1 IFN mechanisms in the context of CHPV pathogenesis. Compared to MOI 2, CHPV infection of WT at MOI 20 led to an early cessation in IFNβ production that paralleled exacerbated cell death (Fig. 1). Despite the impairment of type-1 IFN response, WT cells generated fewer progeny particles at MOI 20. Conversely, enhanced viability of *Nfkbia^−/−^* cells prevented premature waning of IFNβ at MOI 20, and yet these knockout cells supported augmented yield upon raising MOI from 2 to 20 (Fig. 4). These studies indicated that increased cell death at MOI 20 restricted not only viral multiplication but also type-1 IFN response. While emphasizing the mutually exclusive nature of antiviral cellular processes, our results strengthened the notion that type-1 IFNs are less consequential in regulating viral propagation at cell-saturating MOIs where cell death is pervasive. Taken together, we propose that distinct engagement of antiviral processes provides for robust cellular defense at varied MOIs. We also scored a substantive effect of basal type-1 IFN signaling on CHPV growth that was confounded by the NF-κB pathway. It was previously reported that basal IFN signaling is required for boosting signal-induced IFN expressions (48–50). We found that despite heightened virus multiplication, *Ifnar1^−/−^* MEFs were less efficient at IFNβ production upon CHPV infection—a defect that could be attributed to the lack of basal IFN functions in these cells (Fig. 3). Consistent with previous reports (51), constitutive NF-κB signaling in *Nfkbia^−/−^* MEFs promoted basally elevated IFNβ expressions (Fig. 4). We speculate that this basal IFNβ level restricted the frequency of productive infection of *Nfkbia^−/−^* cells at low MOI, lessening CHPV multiplication and virus-induced type-1 IFN response in these mutants. This IFN interference was overcome at very high MOIs (47), presumably leading to an impactful increase in the virus production rate in *Nfkbia^−/−^* cells upon raising MOI from 2 to 20. Future studies ought to combine flow cytometry-based experiments and mathematical analyses and employ compound knockout cells to more comprehensively dissect IFN and cell survival functions of NF-κB signaling in directing the cooperation between antiviral pathways. Similarly, the generalizability of the proposed model needs to be further explored in relation to physiologically relevant cell types.

Previously mathematical model studies improved our understanding of the cellular circuitry driving antiviral responses at varied MOIs and also helped conceptualizing rational therapeutic regimes (52–55). We chose to mathematically analyze host-virus interactions at MOIs of 2 and 20. According to the Poisson distribution, these conditions led to most, if not all, cells being initially infected. Our approach enabled us to specifically examine a uniformly infected population to further understand the interplay between type I IFNs and cell death in constraining viral growth upon increasing input MOI in the cell-saturating high MOI regime. Unlike its predominant role in restricting viral multiplication within already infected cells at high MOI conditions, type I IFNs influence the frequency of new infection during low-MOI infection time courses by inducing an antiviral state in bystander cells. Indeed, a more detailed model incorporating explicit description of uninfected and infected cell compartments, and extracellular virions may offer important insights on viral dynamics at low MOI in the future.

While destroying the viral replicative niche, cell death also orchestrates danger-associated molecular pattern-mediated amplification of pathological inflammation in the infected tissue. Many of the anti-inflammatory drugs, including sodium salicylate and dexamethasone, commonly used for symptomatic management of viral diseases, suppress NF-κB signaling (56–58). We anticipate that the newly described complex interplay between NF-κB functions and input MOIs during the progression of virus infection may bear broader significance for developing therapeutic intervention strategies.

## MATERIALS AND METHODS

### Primary cells and virus

Wild type (WT), *Ifnar1^−/−^* and *Nfkbia^−/−^* MEFs were generated from corresponding 12.5–13.5 day’s embryo and cultured in Dulbecco’s modified Eagle medium (DMEM) reconstituted with 10% BCS. Vero E6 cells were cultured in DMEM supplemented with 10% FBS. The following virus strains were passaged in Vero E6 cells and used in infection studies: CHPV strain 653514 (obtained from the National Institute of Virology, Pune, India) and JEV strain Vellore-P20778.

### Virus infection studies

Semi-confluent culture of primary MEFs were incubated with CHPV and JEV at varying MOI for 1.5 h in serum-free medium. Subsequent to virus adsorption, cells were washed with PBS and the culture was supplemented with DMEM containing 10% BCS. At various times post-infection, either culture supernatants were collected for measuring the titer of CHPV and the abundance of IFNβ or cells were harvested for assessing cell death.

### Virus plaque assay

Culture supernatants collected from infected cells were subjected to serial dilution in the incomplete media. Next, monolayers of Vero E6 cells were infected with these serial dilutions. After 1 h, inocula were removed, and Vero cells were dispensed with the overlay medium containing one part of DMEM and one part of 2% low melting agarose supplemented with 5% FBS at 4°C. Subsequently, cells were incubated at 37°C in a CO2 incubator for another 22 h and then fixed with 10% formaldehyde for 6 h at room temperature. Viral plaques were visualized by staining the fixed VERO E6 monolayer with 0.2% crystal violet for 5 min. Accordingly, viral titer in the culture supernatant was determined as PFU/mL.

### Cell death studies

CHPV-induced cell death was determined by crystal violet staining of adherent, live cells. Uninfected cells were used as controls. Cell death assays were performed using MEFs immortalized using NIH 3T3 protocol.

### Quantification of IFNβ levels

The abundance of IFNβ in the culture supernatant of infected MEFs was determined using a mouse IFNβ ELISA kit (PBL Assay Science, Piscataway, NJ) adhering to manufacturer’s protocol.

### Computational modeling

To mathematically dissect CHPV growth at cell-saturating MOI, a mathematical model consisting of nine parameters was constructed (Fig. 2a, equations i to iii; Table 1). In this model, we assumed that all cells were infected at *t* = 0 [*C*(0) = 1]. We considered that after a time delay of *t*1_delay_, infected cells produced IFN involving a rate constant IFN_pdn_ and progeny viruses were generated involving a rate constant of virus_pdn_. IFN(*t*) and *V*(*t*) represented IFN concentration in pg mL^−1^ and viral titer in pfu mL^−1^ at time *t*, while *C(t)* signified the fraction of infected live cells at time *t* relative to the initial value. As such, the analytical expression *C*(*t*) represented a prescribed phenomenological function. As described below, the computed values of *C*(*t*) were then used in the differential equations governing IFN and virus dynamics (equations ii and iii). The parameter IFN_inh_ represented the rate constant for IFN-mediated inhibition of virus production by infected cells. After an additional delay of *t*2_delay_, cell death occurred involving a rate constant of cell_death_. We considered that a minimum small fraction of cells, *C*_basal_, would remain alive even after prolonged infection; as such, *C*_basal_ did not impact model outputs. Nonetheless, the degradation rate of IFNs and the rate for inactivation of viruses were represented using IFN_deg_ and virus_deg_, respectively. Based on our experiments involving various knockout cells, we also considered a basal IFN level of 2.5 to 5.25 pg/mL in our model.

Briefly, the interferon concentration IFN(*t*) and the extracellular virus titer *V*(*t*) at a given time *t* are governed by the ODEs described in Fig. 2a (equations ii and iii). We conducted a comprehensive literature search to ascribe a range of initial values for various parameters for WT cells (30, 35). We then floated the parameter values. The final values were inferred by minimizing the combined least-square error between the model-predicted virus titers, interferon levels, and the fraction of live cells, and the observed experimental time course data for the MOI of 2 and 20 regimes, as described -


$$\Sigma_{genotypes}\Sigma_{MOIs}\Sigma_{j,t}\frac{(y_{j}(t)-{\hat{y}}_{j}(t){)}^{2}}{y_{j}(t{)}^{2}}$$

where $y_{j}(t)$ is the experimental measurement of variable *j* [*V*(*t*)*,* IFN(*t*)*,* and *C*(*t*)] at time *t* and ${ŷ}_{j}(t)$ is the corresponding model prediction. This formula ensured that variables with larger absolute magnitudes [e.g., *V*(*t*)] did not dominate the fitting process, effectively balancing the contributions of *V*(*t*)*,* IFN(*t*)*,* and *C*(*t*).

Parameters estimation was achieved by minimizing the pooled objective function using Excel’s GRG Nonlinear Solver. Accordingly, a set of parameter values were extracted for the WT settings and also for knockouts, adhering to following assumptions.

- We noted that a rise in MOI from 2 to 20 in WT settings led to a less than 1.25-fold change in *t*1_delay_ from 2.5 h to 2 h; while *t*2_delay_ experienced an insignificant change from 3.25 to 3.24. Accordingly, we assumed that for the scope of our current analyses, *t*1_delay_ and *t*2_delay_ are insensitive MOIs. We then set the values of *t*1_delay_ and *t*2_delay_ in the WT settings at 2.5 and 3.5, respectively. Similar *t*1_delay_ and *t*2_delay_ values were fixed also for other genotypes.
- Because virus growth-inhibitory function and inactivation rate are intrinsic properties of IFNs, we assumed that IFN_inh_ and IFN_deg_*,* are independent of the input MOI and cell genotypes. Accordingly, IFN_inh_ and IFN_deg_ values were kept identical between WT and *Nfkbia^−/−^* systems. For *Ifnar^−/−^* system, IFN_inh_ was set to zero for the want of functional type-1 IFN signaling, and IFN_deg_*,* was kept similar to WT cells.
- Similarly, virus_deg_ and *C*_basal_ were persevered between genotypes.

The system of ODEs was solved numerically using an explicit forward time-stepping scheme implemented in Microsoft Excel. The equations were integrated using a fixed time step (Δ*t*) over the full experimental time window. The time step was chosen to be sufficiently small relative to the fastest dynamical timescale in the system and reducing Δ*t* further did not alter model predictions, confirming numerical stability and convergence. Based on our mathematical analyses, each genotype for a given MOI was assigned a distinct parameter value for virus_pdn_, IFN_pdn_, and cell_death_. These parameter values were extracted based on corresponding experimental time course measurements from three independent biological replicates and accordingly presented as mean ± SEM.

### Statistical analysis

Error bars are presented as standard errors of the means (SEM) of three or more experimental replicates. Statistical significance was determined by Student’s *t*-test for computational studies and by two-way ANOVA followed by Tukey’s multiple comparison for experimental analyses.

### Significance sentence

Cell death—more so than type-1 interferons—limits Chandipura virus propagation at cell-saturating high multiplicity of infection.

### Highlights

Type-1 IFNs and cell death cooperate at varied MOI in limiting CHPV multiplication.

At sub-saturating low MOI, type-1 IFNs play a dominant role in controlling CHPV propagation.

At cell-saturating high MOI, cell death determines the progeny yield.

## Data Availability

All experimental raw data and computational modeling outputs generated during this study are provided in the supplemental material. No custom code was used for data analysis beyond standard spreadsheet operations in Microsoft Excel, as described in Materials and Methods.

## SUPPLEMENTAL MATERIAL

Supplemental legend mbio.01013-26-s0001.docx Caption for the supplemental table.

Table S1 mbio.01013-26-s0002.xlsx Raw experimental and modeling data.

ASM does not own the copyrights to Supplemental Material that may be linked to, or accessed through, an article. The authors have granted ASM a non-exclusive, world-wide license to publish the Supplemental Material files. Please contact the corresponding author directly for reuse.
