# Supplementary material for: A dominant role of cell death in limiting Chandipura virus propagation at cell-saturating high multiplicity of infection
Source: mBio. 2026 Jun 15;17(7):e01013-26. doi: 10.1128/mbio.01013-26 (PMC13343977; doi:10.1128/mbio.01013-26)
Supplement: Supplemental legend — Caption for the supplemental table. [file mbio.01013-26-s0001.docx]

**A dominant role of cell death in limiting Chandipura virus propagation at cell-saturating high multiplicity of infection**

Bhawna^1,2,*^, Swapnava Basu^1,*^, Syed Yusuf Mian^1,3^, Sanchi Arora^1^, Yashika Ratra^1^, Kasturi Ganguly^1^, Sachendra S. Bais^1,4^, Manidipa Banerjee^2^, Abhyudai Singh^5^, Soumen Basak^1,#^

^1^Systems Immunology Laboratory, National Institute of Immunology, Aruna Asaf Ali Marg, New Delhi-110067, India; ^2^Kusuma School of Biological Sciences, IIT Delhi; ^5^Department of Electrical and Computer Engineering, Department of Biomedical Engineering, University of Delaware, Newark, DE, USA

^3^Current address: Harvard T.H. Chan School of Public Health, USA ^4^Current address: Department of Medicine, Washington University School of Medicine, Saint Louis, MO 63130, USA

*these authors contributed equally to this work ^#^corresponding author: email: [sobasak@nii.ac.in](mailto:sobasak@nii.ac.in)

**Supplemental data:**

**The supplementary Excel file contains all the raw data for Figure 1b-1f, 3a-3c, 4a-4c, and 5a-5b, as well as the model-fitting and parameter-estimation files.**
